# Two-Photon Microscopy Imaging of *thy1*GFP-M Transgenic Mice: A Novel Animal Model to Investigate Brain Dendritic Cell Subsets *In Vivo*

**DOI:** 10.1371/journal.pone.0056144

**Published:** 2013-02-07

**Authors:** Claudia Laperchia, Anna L. Allegra Mascaro, Leonardo Sacconi, Anna Andrioli, Alessandro Mattè, Lucia De Franceschi, Gigliola Grassi-Zucconi, Marina Bentivoglio, Mario Buffelli, Francesco S. Pavone

**Affiliations:** 1 Department of Neurological Sciences, University of Verona, Verona, Italy; 2 National Institute of Neuroscience, Verona, Italy; 3 European Laboratory of Non-Linear Spectroscopy, University of Florence, Sesto Fiorentino, Italy; 4 National Institute of Optics, National Research Council, Florence, Italy; 5 Department of Medicine, University of Verona, Verona, Italy; 6 Center for Biomedical Computing, University of Verona, Verona, Italy; 7 Department of Physics, University of Florence, Sesto Fiorentino, Italy; 8 International Center of Computational Neurophotonics, Sesto Fiorentino, Italy; University of Nebraska Medical Center, United States of America

## Abstract

Transgenic mice expressing fluorescent proteins in specific cell populations are widely used for *in vivo* brain studies with two-photon fluorescence (TPF) microscopy. Mice of the *thy1*GFP-M line have been engineered for selective expression of green fluorescent protein (GFP) in neuronal populations. Here, we report that TPF microscopy reveals, at the brain surface of these mice, also motile non-neuronal GFP+ cells. We have analyzed the behavior of these cells *in vivo* and characterized in brain sections their immunophenotype.

With TPF imaging, motile GFP+ cells were found in the meninges, subarachnoid space and upper cortical layers. The striking feature of these cells was their ability to move across the brain parenchyma, exhibiting evident shape changes during their scanning-like motion. In brain sections, GFP+ cells were immunonegative to antigens recognizing motile cells such as migratory neuroblasts, neuronal and glial precursors, mast cells, and fibroblasts. GFP+ non-neuronal cells exhibited instead the characteristic features and immunophenotype (CD11c and major histocompatibility complex molecule class II immunopositivity) of dendritic cells (DCs), and were immunonegative to the microglial marker Iba-1. GFP+ cells were also identified in lymph nodes and blood of *thy1*GFP-M mice, supporting their identity as DCs. Thus, TPF microscopy has here allowed the visualization for the first time of the motile behavior of brain DCs *in situ*. The results indicate that the *thy1*GFP-M mouse line provides a novel animal model for the study of subsets of these professional antigen-presenting cells in the brain. Information on brain DCs is still very limited and imaging in *thy1*GFP-M mice has a great potential for analyses of DC-neuron interaction in normal and pathological conditions.

## Introduction

The generation of transgenic mice expressing fluorescent proteins in subsets of cells in the brain, such as glia, neurons, and monocyte-derived cells, together with the increasing use of two-photon fluorescence (TPF) microscopy have allowed *in vivo* investigations of different cell types at the single cell level [1]–[3]. Thus, advances have been achieved in the investigation of a wide range of phenomena such as dendritic spine remodeling after learning/experience [4]–[7], stroke [8], neuroinflammation [9] and laser dissection [10], [11].

The *thy1*GFP-M knock-in mice express green fluorescent protein (GFP) in about 10% of neurons. In these mice, the regulatory element Thy1 has been modified by deletion of the intron required for the expression of the insert gene in non-neuronal cells [12], [13]. Therefore, *thy1*-expressing non-neuronal cell types (thymocytes, peripheral T cells, myoblasts, epidermal cells, keratinocytes) [14] and dendritic cells (DCs) [15] should not express GFP. However, in the initial description of this mouse line, the occurrence of a few fluorescent-tagged mononucleated cells in the brain was reported as occasional finding, though with no details on their identification and characterization [13].

On the basis of this initial report, we here investigated whether non-neuronal cells, and in particular immune cells, could be detected in the brain of mice of the *thy1*GFP-M line. Our *in vivo* scrutiny of the brain surface of *thy1*GFP-M mice to this purpose was based on TPF microscopy. These analyses have revealed, in the meninges and cerebral cortex, a population of motile GFP-tagged cells which did not exhibit the features of mature neurons. The present study has been focused on the investigation of such GFP-tagged non-neuronal cells at the brain surface of *thy1*GFP-M mice. The cell motile behavior was analyzed through TPF imaging *in vivo* followed by immunohistochemical phenotyping in brain sections. Since the findings revealed the identity of these cells as immune elements, blood and lymph nodes were also examined.

## Materials and Methods

### Animals

Young adult (3–6 month-old) *thy1*GFP-M and C57BL6J (wild-type, WT) and L7-GFP mice were used. A colony was established in the animal facilities at the Medical Faculty of the University of Verona from breeding couples (kindly supplied by Dr. Joshua Sanes, Harvard University, Boston, MA, USA). Mice were genotyped by polymerase chain reaction (PCR) using the e-YFP-F1 and e-YFP-R4 primers (eYFPF1: 5'ATCTTCTTCAAGGACGACGGCAACTACAAG3'; eYFPR4: 5'AGAGTGATCCCGGCGGCGGTCACGAACTCC 3'). The amplification product was separated by 1.8% agarose gel electrophoresis and visualized by Syber Green staining (Sigma, Milan, Italy) under UV light.

The animals were maintained under standard environmental conditions (temperature, humidity, 12 h/12 h light/dark cycle, with water and food *ad libitum*) under veterinarian assistance. Animals handling and surgery were performed following a protocol which received approval by the Animal Care and Use Committee of the University of Verona (CIRSAL), and authorization by the Italian Ministry of Health, in strict adherence to the European Communities Council (86/609/EEC) directives, minimizing the number of animals used and avoiding their suffering.

### In vivo *imaging*

For *in vivo* imaging the open skull technique was performed as previously described [16]. Briefly, the mice were deeply anesthetized by ip injection of ketamine (90 mg/kg) and xylazine (9 mg/kg). A low dose of dexamethasone (0.04 ml at 2 mg/ml) was administered prior to surgery to minimize brain swelling. The animals were then placed on a stereotaxic frame; a heating blanket was used to prevent hypothermia and the eyes were protected from dehydration by a drop of saline.

For the open skull technique, a small craniotomy was performed under the dissecting microscope by delimiting with a dental drill an area of about of 25 mm^2^, while the skull was frequently refreshed by application of a drop of saline. The bone flap was removed and a circular cover glass was applied to cover the dura and sealed to the skull by cyanoacrylate mixed with dental acrylic cement.

In 4 mice, superficial blood vessels were labeled with the red fluorescent dye sulforhodamine 101 (SR101) by a brief application of a 500 nM solution on the cortex before placing the optical window [17]. In 6 mice, blood plasma was labeled through tail vein injection of a 0.2-ml bolus of 5% (w/v) either Texas Red dextran (70 kDa) (Invitrogen, Milan, Italy; D-1830) or tetramethyl rhodamine isothiocyanate-conjugated dextran in saline [18], [19]. Control experiments (n  =  3) were also performed using the thinned skull technique as described by Yang et al. [20].

At the end of surgery, the mice were woken up and left on the heating blanket until recovery; dehydration was prevented by subcutaneous injection of saline. The animals then received antibiotic treatment (enrofloxacin, 5 g/kg, ip), and were returned to their home cage for at least 24 h after surgery. To minimize inflammatory phenomena that may occur after surgery, the mice were treated daily with the anti-inflammatory drug Carprofen (5 mg/kg, sc).

TPF imaging was performed using a custom-made, upright, scanning microscope as previously described [10], [11] or through a Leica SP5 microscope equipped with a pulsed Ti: sapphire laser (Chameleon, Coherent Incorporated, Santa Clara, CA) and with an objective lens Leica HCX APO L20x/NA0.95, water immersion. A detection system (PML-Spec, Becker &Hickl GmbH, Berlin, Germany) constituted by a diffraction grating and a 16-channels multi-anode photomultiplier strip was used to acquire the fluorescence spectrum. This allows spectral resolved (13 nm for each channel) measurements of fluorescence light with variable spectral range.

### Analysis of *in vivo* imaging data

*In vivo* TPF 3D stacks were analyzed through an open source imaging processing software (ImageJ) and Imaris software (BitPlane, Zurich, Switzerland). The Spot Analysis was used for semi-automated tracking of cell motility in three dimensions over time. For cell speed, the coordinates of each cell were calculated and tracked over time. Since motion artifacts can be caused by dendrite “probing”, animal’s pulse and breathing, displacements smaller than 2.0 µm/min were filtered out of the cell tracks [21]. Cells showing a displacement below the threshold of 2.0 m/min were therefore considered as static (sessile) cells.

### Histology, immunohistochemistry and confocal microscopy on brain and cervical lymph node sections

For the *ex vivo* study, *thy1*GFP-M mice (n  =  7) were deeply anesthetized (trybromethanol, 1.25% final solution; 20 mg/kg, ip) and transcardially perfused with 0.1 M phosphate-buffered saline, pH 7.2 (PBS), followed by 4% paraformaldehyde in PBS. The brain and superficial cervical lymph nodes were rapidly dissected out and stored for cryoprotection in 30% sucrose in PBS at 4^°^C until they sank. The tissue samples were then embedded in OCT cryoembedding matrix and sectioned on the coronal plane at a 30 µm thickness (brains) or 20 µm thickness (lymph nodes) with a cryostat or a freezing microtome.

For the immunohistochemical procedure, brain and lymph node sections were first treated for 20 min at room temperature with a blocking solution of 2% bovine serum albumin (BSA), 2% normal goat serum or fetal serum and 0.2% Triton X100 in PBS, and then incubated overnight at 4^o^C in primary antibodies. Brain sections were processed with the primary antibodies listed in Table 1. Lymph node sections were processed for immunohistochemistry with CD11c and/or CD3 primary antibodies (Table 1). After washes in PBS, sections from all the tissue samples were incubated with secondary antibodies for 2 h at room temperature. The following secondary antibodies (all purchased from Invitrogen) were used: anti-rat IgG Alexa 594; goat anti-hamster IgG Alexa 568; goat anti-mouse IgG Alexa 549; goat anti-mouse IgG Alexa 647; goat anti IgGM 350; anti IgG1 Alexa 647; anti-rabbit IgG (H+L) Alexa 568; anti-rat IgG 594, and anti-mouse IgG1 Alexa 594. Secondary antibodies were all diluted 1∶1000 in the above blocking solution, with the appropriate serum. After immunohistochemical processing, the sections were counterstained with the fluorescent nuclear marker DAPI (4’–6’-diamidino-2-phenylindole) and mounted on slides with 0.1% paraphenylendiamine in glycerol-based medium (90% glycerol and 10% PBS).

**Table 1:** Immunophenotypic profile of NeuN-/GFP+ cells in *thy1*GFP-M mice.

ANTIBODY	SUPPLIER	IMMUNOGEN, DILUTION		ANTIGEN	CELL TYPE	NEuN-/GFP+ CELLS
*NEURONAL AND GLIAL PROGENITOR*						
anti-CD133	Santa Cruz *Santa Cruz,CA*	rabbit IgG 1∶500		prominin	quiescent neural stemcells	–
anti-NG2	Chemicon *Temecula,CA*	rat IgG 1∶500		chondroitinsulfate proteoglycan NG2	oligodendrocyte; neural progenitors	–
anti-O4	Millipore *Billerica,MA*	mouse IgM 1∶500		O4	pro-oligodendrocytes	–
anti-A2B5	Millipore *Billerica, MA*	mouse IgM 1∶500		A2B5	oligodendrocyte progenitors	–
anti-nestin	Chemicon *Temecula,CA*	mouse IgG11∶200		nestin	neural stemcells	–
*NEUROBLASTS*						
anti-DCx	Santa Cruz *Santa Cruz,CA*	goat IgG 1∶100		doublecortin	migratory neuroblasts	–
anti- TUJ-1	Covance *Princeton,NJ*	mouse IgG2a 1∶500		neuronal class III-tubulin	neuroblasts	–
*NEURONS*						
anti-NEuN	Chemicon *Temecula,CA*	mouse IgG1 1∶200		neuronal nuclei protein	neurons	–
anti-GAD-67	Chemicon *Temecula,CA*	mouse IgG1 1∶1000		glutamic acid decarboxylase 67	GABAergic neurons	–
anti-MAP-2	Sigma *Saint Louis, MO*	mouse IgG1 1∶1000		microtubulo associated protein-2	neuronal dendrites	–
*ASTROCYTES*						
anti-GFAP	Chemicon *Temecula,CA*	mouse IgG1 1∶500		glial fibrillary acidicprotein	astrocytes	–
*ANTIGEN-PRESENTING CELLS*						
anti-MHC-II	AbDSerotec *Oxford, UK*	mouse IgG1 1∶500	major histocompatibility complex class II		dendritic cells, microglia, macrophages	+^A^
anti-CD11c	Biolegend *San Diego CA*	armenian hamsterIgG 1∶500	complement type 4 receptor (CR4)		subset of dendritic cells, microglia	+^A^
anti-Iba-1	Santa Cruz *Santa Cruz,CA*	rabbit IgG 1∶500	ionized calcium binding adaptor molecule 1		microglia, monocytes, neutrophils	–
anti-CD11b	AbDSerotec *Oxford UK*	rat IgG2b 1∶500	complement type 3 receptor (CR3)		subset of dendritic cells, microglia, subset of macrophages, natural killer cells	+^B^
anti-F4/80	Acris antibodies *Herdford, Germany*	rat IgG2b 1∶200	F4/80		macrophages	–
*OTHER NON-NEURONAL CELLS*						
anti-avidin	Chemicon *Temecula,CA*	mouseIgG 1∶500		avidin	mastcells	–
anti-fibronectin	Acris antibodies *Herdford, Germany*	mouse IgG1 1∶500		fibronectin	fibroblasts	–

A Subset of positive cells in higher number in meninges than in choroid plexus ;^B^Subset of positive cells mainly round shaped and without ramifications.

Preliminary observations were performed to detect GFP+ non-neuronal cells on sections from mice sacrificed after *in vivo* imaging and processed (as described above) for double immunostaining antibodies. Anti-laminin was used to visualize the pia mater, and anti-NeuN antibody to discriminate GFP+ neurons from GFP+ non-neuronal cells. NeuN-/GFP+ cells were identified at the same location and with the same morphology observed *in vivo*.

For immunohistochemical analysis and cell counts, coronal brain sections were sampled from bregma 1.18 to bregma -2.70 (stereotaxic coordinates from [22]), collected in adjacent series of every fourth section. At least 75% of pia mater extent and the choroid plexus were preserved in the collected sections, which were otherwise discarded. To quantify the percentage of non-neuronal GFP+ cells with respect to the total amount of major histocompatibility complex class II (MHCII+) and CD11c+ cells, a total of 12 brain sections were collected from four *thy1*GFP-M mice. Analysis of the percentage of non-neuronal GFP+ cells expressing MHCII and/or CD11c was performed on 18 coronal sections from six brains. Cell counting was performed scanning each section with the confocal microscope Leica SP5 (Leica, Manheim, Germany) and 0.8 µm serial Z-stacks digital photographs were captured and analyzed for the colocalization of the above markers and GFP with the software Leica Application Suite (LAS).

Image acquisition was performed with the confocal microscope Leica SP5. Serial Z-stack digital images (0.8 µm) were captured and analyzed with Leica Application Suite software.

### Flow cytometry analysis on whole blood

Blood samples were collected by retro-orbital venipuncture in anesthetized mice using heparinized microcapillary tubes and used for flow cytometric analysis as previously described [23]. Briefly, red blood cells were lysed in lysis buffer (155 mM NH_4_Cl, 10 mM KHCO_3_, 5% EDTA pH 7.4) 1∶10 v/v for 10 minutes. White blood cells were washed once with 1% BSA in phosphate buffer and incubated in 1% BSA in PBS with anti-mouse CD16/CD32 (Clone 2.4G2, BD Biosciences, San José, CA, USA) for 15 min, to block Fc receptors and reduce nonspecific binding. Samples were stained with the anti GR-1-APC (Ly-6G/Ly-6C, Clone RB6-8C5, BD Biosciences, Mountain View, CA), CD11b APC-Cy7 (Clone M1/70, BD Biosciences), CD8a PerCP-Cy5.5 (Clone 53-6.7, eBioscience, San Diego, CA USA), CD4 PE-Cy7 (Clone GK1.5, eBioscience), CD3 APC (Clone 17A2, eBioscience), CD45R/B220 APC-Cy7 (Clone RA3-6B2, BD Biosciences), F4/80 Pe-Cy7 (Clone BM8, eBioscience). All phenotypic assays of WT and *thy1*GFP-M samples were performed in parallel. Flow cytometric analysis was performed on a FACS Canto flow cytometer (BD Biosciences) and data were analyzed by FlowJo software version 7.6.4 (Tree Star, Ashland, OR, USA). Different cell populations were clustered according to the presence and intensity of cell surface epitope markers [23]–[25]. Debris and doublets were excluded from the analysis based on the Side/Forward light scattering plot and FSC-H/FSC-A plot respectively (data not shown). GFP+ cells were arbitrarily set up to about 0.1% of the highest negative control and then applied to all the other samples, to include the vast majority of GFP+ cells in *thy1*GFP-M blood. Single stains were used as compensation controls.

## Results

### *In vivo* observation of GFP+ cells in the meninges and at the cortical surface of *thy1*GFP-M mice

TPF imaging was performed in adult *thy1*GFP-M mice (n  =  12) through the optical window as described above. With this approach, the apical dendrites of GFP-tagged neurons, their dendritic spines and apical tufts were visualized in cortical layers I and II, together with cell bodies of pyramidal neurons residing in layers III and neuronal elements in layer IV.

**Figure 1:**
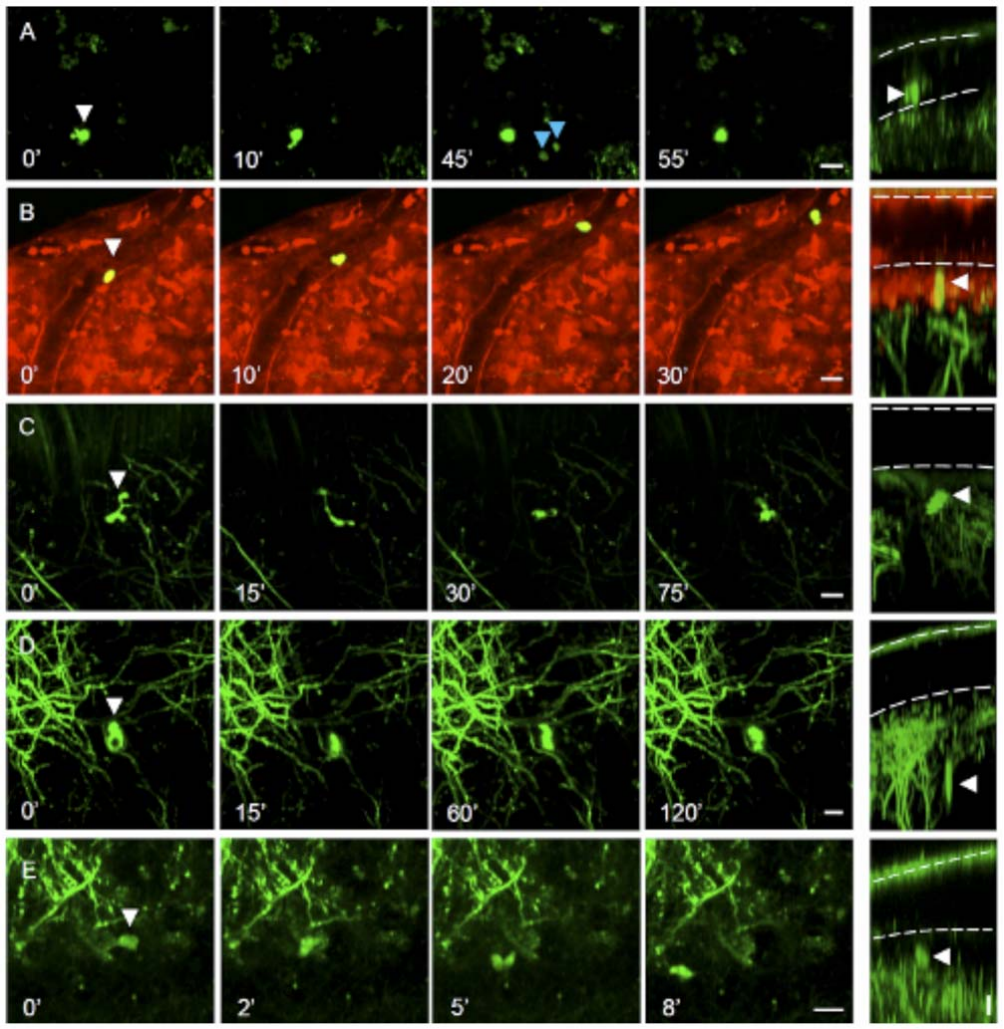
*In vivo* observation of motile GFP-labeled cells in the cortex of *thy1*GFP-M mice. (A) GFP+ cell (white arrowhead) above the pial surface rapidly changing its morphology. Time-lapse sequence of maximum intensity projections of a set of optical sections acquired at 2 µm z-step. The right column shows the depth of the cell through a digital rotation of the corresponding images on the left. The white dotted lines indicate the dura and pia mater.The frame acquired at 45′ shows fluorescent cells (blue arrowheads) passing above the pia through the CSF. (B) GFP+ cell rolling inside a blood vessel on the pial surface. The blood vessel walls (shown in red) were labeled by the intravital dye SR101. Fluorescent cells showing motility at the pial surface (C) and deep in the brain parenchyma (D–E). E) GFP+ cells showing translation across the field of view. Scale bars 10 µm.

Motile green fluorescent cells with morphological features and distribution different from those of neurons were also observed in the TPF microscopy investigation (Figure 1). To clarify whether the fluorescence emission of these non-neuronal cells derived from the same fluorescent protein expressed in neurons (GFP), we characterized *in vivo* the emission spectrum of the fluorescent non-neuronal cells through a multi-channel acquisition. We found that the fluorescence spectrum of green non-neuronal cells was identical to the spectrum of GFP-labeled neurons (see Figure S1). This finding suggests that the fluorescence expression in GFP-labeled non neuronal cells may be under the control of the same promoter (Thy-1) that targets neurons in *thy1*GFP-M mice [13]. In agreement with this hypothesis, we found that in L7-GFP mice expressing GFP on Purkinje cells under a different promoter [26], no fluorescent motile cells were detected during TPF imaging experiments (n  =  25).

The time-lapse TPF analysis in *thy1*GFP-M mice revealed that the size and motility of GFP+ non-neuronal cells were different depending on the region. At the most superficial level (0–15 µm depth from the dura), in the subarachnoid space, round-shaped GFP+ cells with a diameter of 9.9 ± 1.3 µm (number of cells  =  7; number of mice  =  4) were observed (Figure 1A and Movie S1) laying on the pia mater or floating in the CSF. At the same depth, these cells were found inside meningeal blood vessels as well, passively transported in the blood stream; GFP+ cells rolling on the endothelial surface of blood vessel walls were also occasionally observed (Figure 1B and Movie S2).

At the interface between pia mater and brain parenchyma (depth: 15–25 µm), most of the non-neuronal GFP+ cells were round-shaped (diameter of 8.0 ± 1.0 µm; number of cells  =  12; number of mice  =  6) and static with average displacements smaller than 2.0 µm/min. In this region a few GFP+ non-neuronal cells exhibited an irregular shape and a dynamic behavior. Most of the GFP+ cells observed within the cortical tissue (depth: 25–60 µm from the pia) were characterized by fast remodeling of their shape (in a time scale of minutes), with continuous extensions and retractions of their processes (Figure 1). Changes in morphology were accompanied in some instances by translation of the center of the cell body mass with displacement speeds higher than 2.0 µm/min (Figure 1 E and Movie S3). This motile behavior appeared related to a scanning motion in the brain parenchyma. The thinned skull preparation surgery, a less invasive surgical approach in which the bone flap is not removed but just reduced to a thin layer, confirmed the presence of GFP+ non-neuronal cells which exhibited the same motile behavior observed through the chronic cranial window (Movie S4). This indicates that in the open skull window preparation the occurrence of GFP+ motile cells was not related to potential inflammatory responses to the surgical approach.

### Phenotypic and quantitative characterization of GFP+ cells in the meninges and choroid plexus of *thy1*GFP-M mice

**Figure 2:**
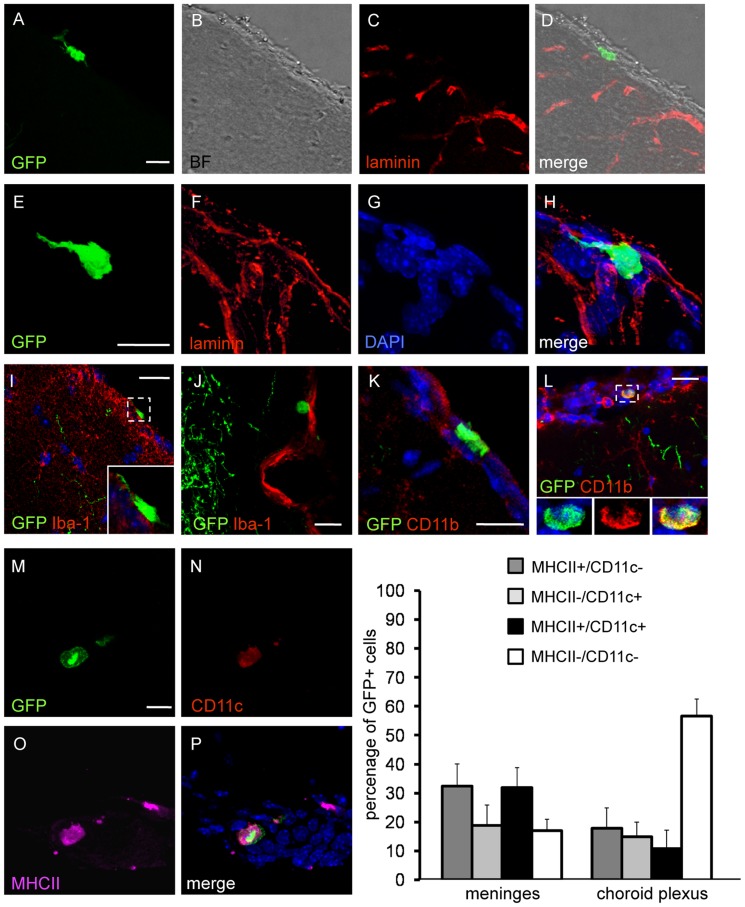
Distribution of the NeuN-/GFP+ cells in the brain of *thy1*GFP-M mice and quantitative analyses . Confocal microscopy images of brain sections showing non-neuronal GFP+ cells with different morphologies in the meninges. (A–D) Ramified non-neuronal GFP+ cell at the pia/parenchyma interface visualized in bright-field in B. (E–H) Elongated non-neuronal GFP+ cell in the subarachnoid space; blood vessels and pia mater were visualized by anti-laminin immunostaining (F, red). (I–J) Non-neuronal/GFP+ cells with ramifications or round-shaped (J), do not express the microglial marker Iba-1 (red). The insert in I represents an high magnification of the area in the dashed box. (K–L) The glial/monocyte marker CD11b (red) was not expressed by ramified cells in the leptomeninges (K) but is instead expressed by small-size round cells (example shown in L), which are therefore identified as monocytes. (M–P) A non-neuronal GFP+ cell in the meninges covering the optic tract (cell nuclei are stained with DAPI, blue). This cell exhibits the morphological features of a migratory dendritic cell, whose cytoplasmic organization in a “veil-like” structure provides a more efficient motility (veiled cell); the immunopositivity to dendritic markers CD11c (N, red) and major histocompatibility complex class II (O, magenta), together with its morphology, confirms the identity of dendritic cell. Scale bars 20 µm. The graph shows the percentage of GFP+ cells immunopositive to anti-MHCII, anti CD11c or both these markers. Data were obtained from 83 and 118 of GFP+ cells counted in 6 animals in meninges and choroid plexus, respectively.

The phenotype of GFP+ cells and their distribution were analyzed in fixed brain sections, thus including regions not accessible during *in vivo* imaging sessions. GFP+ cells were distinguished from neurons on the basis of their immunonegativity to the antibody against neuron-specific protein NeuN (Table 1). The pia mater and blood vessel walls were visualized by laminin immunofluorescence or under bright-field observation to ascertain the distribution of NeuN-/GFP+ cells with respect to these structures. In line with *in vivo* observations, NeuN-/GFP+ cells were observed along the meninges covering the cerebral cortex, at the pia/cortical parenchyma interface (Figure 2 A–D) as well as above the pia mater, in the subarachnoid space (Figure 2 E–H). These cells were mostly associated with meningeal blood vessels, in the perivascular space or in close association with the endothelium. NeuN-/GFP+ cells were also found in the meninges covering the interhemispheric fissure and in the choroid plexus. With the exception of cells within blood vessels and in the choroid plexus, which exhibited round/ovoid shape (8–14 µm in diameter), most NeuN-/GFP+ cells showed an elongated shape, with a long axis ranging from 16 µm to 22 µm.

The dynamic behavior of these cells documented in the *in vivo* experiments led us to investigate their immunophenotype with markers recognizing motile cells in the adult brain (see Table 1). Confocal microscopy analyses on cryosections ruled out the nature of these cells as migratory neuroblasts, stem cells, neuronal and glial common progenitors. Furthermore, NeuN-/GFP+ cells were immunonegative to avidin and fibronectin, markers for mast cells and fibroblasts, respectively, which are also able to move in the brain or at its surface. NeuN-/GFP+ cells did not express positivity to glial fibrillary acidic protein, which ruled out their identity as astrocytes. Moreover, these cells were immunonegative for the microglial/macrophages antigen Iba-1, regardless of their localization and morphology (Figure 2 I–J). NeuN-/GFP+ cells were occasionally observed (about one cell per section) in the meninges. They exhibited a diameter ranging from 5 to 9 µm and immunopositivity to the CD11b antigen, expressed not only by microglial cells but also by neutrophils and monocytes (Figure 2 K–L). The majority (more than 60%) of the NeuN-/GFP+ cells identified in the meninges expressed MHC II, thus indicating that they were antigen-presenting cells (Figure 2 M–P and graph). On this basis, markers of macrophages and DCs, which can act as motile antigen-presenting cells in the brain, were tested (see Table 1). NeuN-/GFP+ cells in the meninges and choroid plexus did not express F4/80, the most common marker of macrophages [27], and expressed CD11c, a marker for DCs [28].

Taken together these results indicate that the immunophenotype of NeuN-/GFP+ cells was MHCII+/CD11c+/CD11b+/Iba1-/F4/80-, and they could therefore be identified as DCs. Importantly, in agreement with this characterization, we also occasionally observed GFP+/CD11c+/MHCII+ cells with a cytoskeletal arrangement typical of migratory DCs (veiled cells, Figure 2 M–P) [29].

Cell counts were performed in the meninges and choroid plexus, where NeuN-/GFP+ cells were mostly observed, to evaluate the proportion of GFP+ DCs with respect to the entire DCs population (MHCII+/CD11c+) and the proportion of GFP+ cells expressing MHCII or CD11c, respectively. This part of the study indicated that GFP+ DCs represented 10.30 ± 3.85% of DCs in the meninges and 13.08 ± 4.30% of DCs in the choroid plexus. With respect to either marker, in the meninges GFP+/MHCII+ cells accounted for 7.12 ± 0.21% of the MHCII+ cell population, and GFP+/CD11c+ cells for 9.19 ± 3.15% of the CD11c+ cell population. In the choroid plexus, GFP+/MHCII+ represented 6.80 ± 1.61% of MHCII+ cells, and GFP+/CD11c represented 15.04 ± 8.32% of CD11c+ cells.

Quantitative analysis of the phenotype of GFP+ cells was then pursued. In the meninges about 83% of GFP+ cells were identified by the expression of MHCII and/or CD11c (GFP+/MHCII+/CD11c+ 31.82 ± 6.88%; GFP+/MHCII+/CD11c- 32.37 ± 7.66%; GFP+/MHCII-/CD11c+ 18.71± 7.15%) (Figure 2 Q). In the choroid plexus, about 40% of GFP+ cells were identified with the same markers (GFP+/MHCII+/CD11c+ 10.75 ± 6.48%; GFP+/MHCII+/CD11c- 17.77± 7.02%; GFP+/MHCII-/CD11c+ 14.81± 5.16) (Figure 2 Q).

### Distribution of NeuN-/GFP+ cells in the brain of *thy1*GFP-M mice

**Figure 3:**
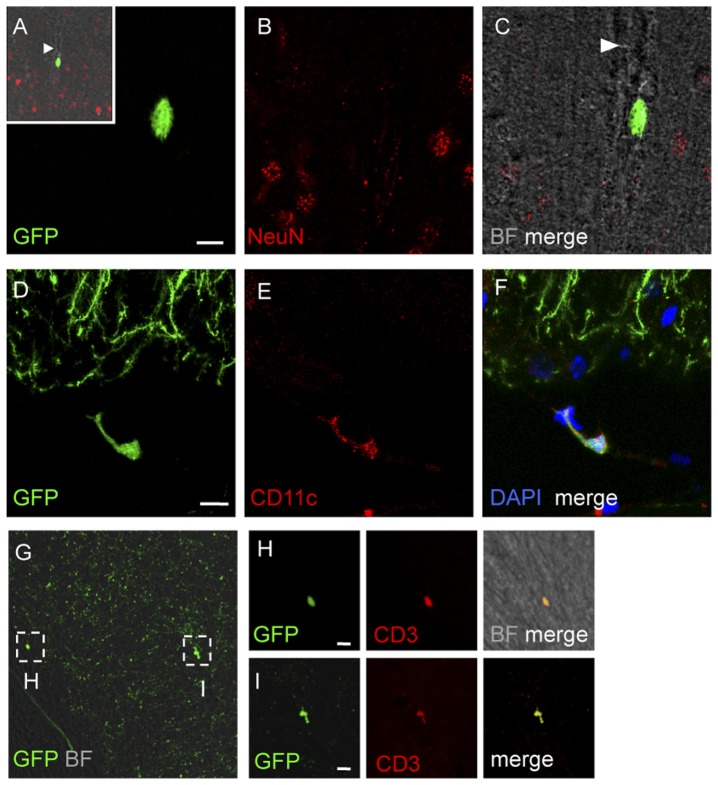
Immunophenotypic analysis of the non-neuronal GFP-tagged cells in the brain of *thy*GFP-M mice. Confocal images of coronal brain sections showing non-neuronal GFP+ cells in several locations. (A–C) GFP+ cell into the lumen of a blood vessel (arrowhead) in layer II of the parietal cortex. Neuronal nuclei were stained with anti-NeuN, (B, C, red); the blood vessel is visualized in bright-field (insert A, C). The inset in A represents the area shown an high magnification in A–C. (D–F) GFP+ cell in the ependyma between hippocampus and thalamus. This cell is immunopositive to the dendritic cell marker anti-CD11c (E, red). Cell nuclei are stained with DAPI in F and represented in blue. (G–I) In the anterior hypothalamus a high number of small-sized GFP+ cells shows immunopositivity to the lymphocytes marker CD3+. Different morphologies of GFP+/CD3+ cells in the anterior hypothalamus. A small round (H, high magnification) GFP+/CD3+ cell and a GFP+/CD3+ cell showing an irregular shape (I, high magnification). Scale bars 10 µm.

At the examination in confocal microscopy, NeuN-/GFP+ cells with different morphologies and sizes were observed in the brain parenchyma. In layers I and II of the parietal cortex NeuN-/GFP+ cells with thin ramifications were found to be associated with the abluminal side of blood vessel walls. In the same area, a few round-shaped cells, 6–8 µm in diameter, were observed inside the lumen of blood vessels (Figure 3 A-C). NeuN-/GFP+ cells were observed in layer II of the piriform cortex and in the corpus callosum and in circumventricular organs. In particular, in the subfornical organGFP+ cells exhibited a peculiar morphology, with elongated/stellate or elongated/dendriform shape and multiple ramifications. Ramified NeuN-/GFP+ cells were also found in the subventricular zone and in the molecular layer of the hippocampus. In the ependyma, a few GFP+ cells were identified (Figure 3 D–F).

In addition, GFP-labeled NeuN- cells intensely GFP-labeled were observed in the medial thalamus and in the anterior hypothalamus (in particular in the premammillary nucleus), and in the arcuate nucleus. Only in these regions, most of NeuN-/GFP+ cells were CD3 immunopositive, without ramifications and very small (3–6 µm in diameter), indicating their nature of lymphocytes (Figure 3 G–I).

### GFP+ cells are present in the blood and cervical lymph nodes of *thy1*GFP-M mice

To further confirm the identity of NeuN-/GFP+/MHC-II+/CD11c+/CD11b+/Iba1-/F4-80- cells as DCs, the occurrence of these cells was verified in anatomical districts in which DCs are normally present, i.e. blood and cervical lymph nodes.

**Figure 4:**
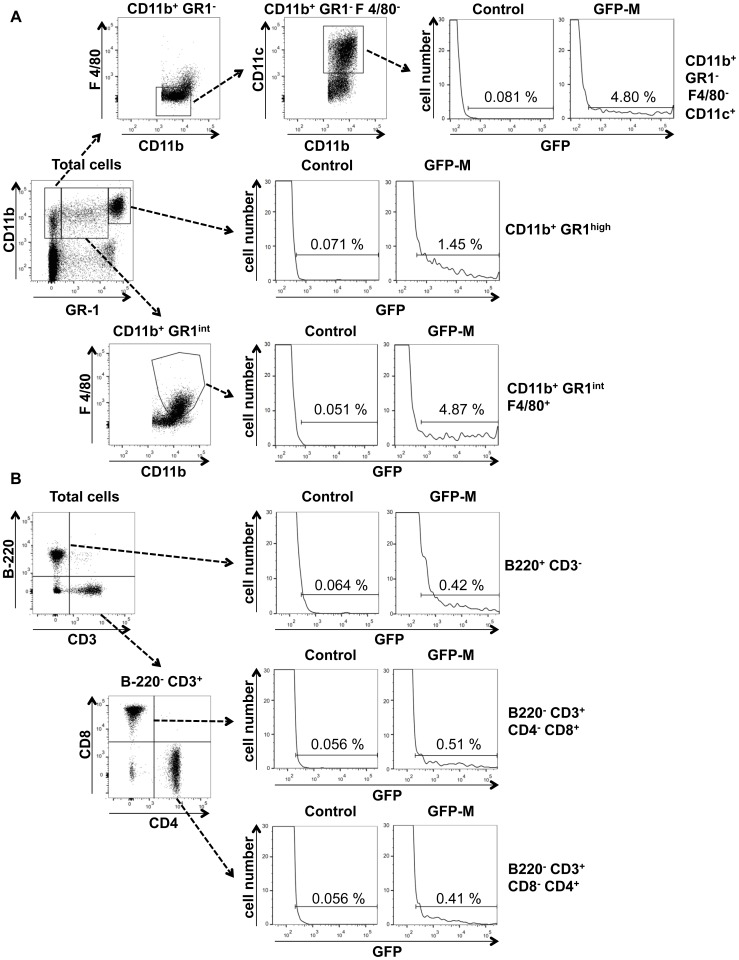
Cytofluorimetric analysis of GFP expression in blood cells from WT and *thy1*GFP-M mice. (A) GFP expression was evaluated in monocytes as CD11b positive, GR-1 (Ly6C, Ly6G) intermediate, F4/80 positive cells (CD11b^+^ GR1^int^ F4/80^+^); in granulocytes as CD11b positive, GR-1 highly positive cells (CD11b^+^ GR1^high^); in dendritic cells as CD11b positive, GR-1 negative, F4/80 negative, CD11c positive cells (CD11b^+^ GR1^-^ F4/80^-^ CD11c^+^). (B) Lymphocyte population were phenotypically characteryzed as B220 positive, CD3 negative cells (B lymphocytes, B220^+^ CD3^-^); B220 negative, CD3 positive, CD8 positive, CD4 negative cells (T cytotoxic cells, B220^-^ CD3^+^ CD8^+^ CD4^-^) and B220 negative, CD3 positive, CD8 negative, CD4 positive (T helper, B220^-^ CD3^+^ CD8^-^ CD4^+^). Percentages inside the gates represent positive cells of parent populations. The figures shown are a representative experiment of 3 other separate experiments from 3 different mice performed with similar results.

GFP expression was evaluated on blood cells by FACS analysis. We found that 1.23 ± 0.18% of the CD11b-positive, GR-1 high positive cells (CD11b^+^GR-1^high^, granulocytes) were GFP-positive (Figure 4 A). To detect monocytes and myeloid DCs, we used a panel of antibodies to better cluster the two cell types, as previously reported [23], [24]. Monocytes are CD11b-positive, F4/80-low positive and low/intermediate positive for GR-1 (CD11b^+^ GR-1^int^ F4/80^+^) [23] while myeloid DCs are CD11b-positive, CD11c-positive and F4/80-negative, GR-1-negative (CD11b^+^ CD11c^+^GR-1^-^F4/80^-^ Figure 4 A) [24]. We found 3.13 ± 1.52% monocytes and 3.19 ± 1.49% DCs GFP-positive (Figure 4 A).

We then evaluated GFP positive cells in the lymphocyte populations [25]. We found 0.34 ± 0.21% of the B220-positive, CD3-negative population (B220^+^CD3^-^, B lymphocytes), 0.34 ± 0.11% of the CD3, CD4-positive, B220, CD8-negative population (CD3^+^B220^+^CD4^+^CD8^-^, T Helper) and 0.59 ± 0.16% of the CD3, CD8-positive, B220, CD4-negative population (CD3^+^B220^-^CD4^-^CD8^+^, T Cytotoxic) (Figure 4 B). As shown in Figure 4 the distribution of GFP fluorescence span over three order of magnitude of intensity suggesting different levels of expression in blood cells [30].

**Figure 5:**
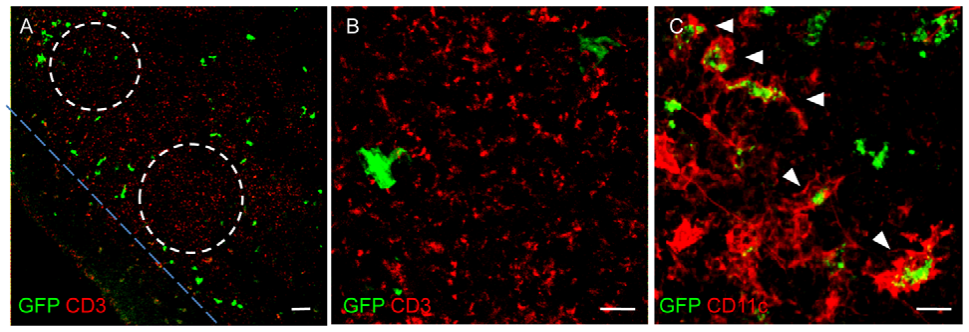
GFP-DCs in cervical lymph nodes of a *thy1*GFP-M mouse. (A) Confocal analysis of cryosectioned cervical lymph node shows the presence of numerous GFP-tagged cells (green). They surround the B cell follicles (dashed lines) and extend in the T cell zone (on the upper right of the figure), while they rarely occur in the subcapsular zone. CD3+ cells, visualized by immunohistochemistry, are here shown in red. (B) High magnification of CD3+ cells (red) and GFP+ cells (green) in the T cell zone. (C) Immunopositivity of GFP-tagged cells (green) to CD11c (red; white arrowheads). Note that the GFP is expressed in the cytoplasm of GFP+DCs.

GFP-labeled cells were found in lymph nodes (Figure 5), where they exhibited the typical morphological features of DCs, with long membrane extensions, and were mainly observed around the B cell follicles and extended into the T cell zone, rarely occurring in the subcapsular zone (Figure 5 A). They showed immunopositivity to anti-CD11c, which thus confirmed their identity as DCs (Figure 5 C).

## Discussion

The present investigation shows that in *thy1*GFP-M mice, engineered to express GFP in neurons, a GFP-tagged monocyte-derived cell population is observed in the meninges, perivascular space, choroid plexus, as well as in brain tissue. These cells show the morphological features, motile behavior, and antigen expression which characterize DCs, and are Iba-1-immunonegative and therefore distinct from microglial cells. GFP+ DCs have also been here identified in cervical lymph nodes and blood of *thy1*GFP-M mice, in agreement with their identity as DCs.

Our imaging analysis with TFP has allowed detection of GFP-tagged brain DCs in *thy1*GFP-M mice *in vivo* and we here show for the first time their motile behavior. Such sets of data are discussed below.

### Brain dendritic cells visualized *in vivo* in *thy1*GFP-M mice

DCs are professional antigen-presenting cells which modulate the delicate balance between induction and suppression of immune response by self or no-self antigen presentation to T cells [31], [32]. DCs are equipped with the most potent antigen-presenting capacity among all immune cells [33].

DCs are well characterized in peripheral organs, especially in lymph nodes, where antigen presentation mainly occurs. In the CNS, these cells are strategically located at the interface between blood/cerebral parenchyma (subarachnoid space), CSF/cerebral parenchyma (meninges, ependyma), CSF/blood (choroid plexus), suggesting an immunosurveillance function for brain DCs as well [34]. However, their role in the immune-specialized CNS remains elusive and, as recently emphasized, DCs are the least explored among leukocytes which can be recruited to the CNS [33].

*In vivo* imaging has here allowed the observation for the first time of the motile behavior of brain DCs *in situ*. They were found to be mainly sessile in the meninges, and in probing motion in the cortical parenchyma. We could thus observe GFP-DCs in a scanning-like motion in vascular-rich brain regions, at the brain surface of *thy1*GFP-M mice, in agreement with a role of immunosurveillance of the healthy brain [31], [34], [35]. A similar motile behavior has been previously described *in vivo* by TPF microscopy in inguinal lymph nodes of healthy eYFP-CD11c mice, in which CD11c is tagged with the enhanced yellow fluorescent protein [21].

It has been shown in healthy rats that DCs, after intracerebroventricular injection, can reach the cervical lymph nodes via the CSF. Therefore, the CSF represents the major transport route for DCs circulating in the CNS and migrating either from CSF to brain or from CSF to lymphoid organs in physiological conditions [36]. In agreement with these findings, in *thy1*GFP-M mice GFP+/CD11c+ cells also reside in cervical lymph nodes. In addition, migratory DCs can undergo evident cytoskeletal rearrangement for efficient motility to cervical lymph nodes, and are therefore indicated as veiled cells [29]. It is interesting to note that we here observed this peculiar morphology of GFP+ cells in the meninges of *thy1*GFP-M mice.

### Characterization of DCs

DCs are a heterogeneous class of cells and their identification and distinction from other cell types, in particular from macrophages, is highly debated [37]. Indeed, myeloid lineage markers, co-stimulatory molecules and antigens expressed by DCs recognize only subsets of these cells and/or are expressed also from other cell types [38]. It has also been proposed that DCs and macrophages represent the continuum of a common progenitor [39].

The most widely used marker for DCs is currently provided by CD11c, which, however, is not sufficient for unequivocal identification of these cells (see for review [37], [40]). Thus, a multiple immunophenotyping approach is required to identify DCs. Our multilabeling strategy has shown that the non-neuronal GFP-tagged cells which occur in the meninges of *thy1*GFP-M mice are CD11c+/CD11b+/MHC-II+/F4/80- and can therefore be identified as DCs on the basis of their currently accepted immunophenotype [37].

The quantitative analysis in the meninges and choroid plexus of *thy1*GFP-M mice has revealed heterogeneity in the expression of MHCII and CD11c markers in DCs. Such heterogeneity was also shown in previous studies of DCs in the rat choroid plexus [41] and in whole mount preparations of the rat meninges [42], [43].

Quantitative analyses of putative DCs in the meninges and choroid plexus have been recently reported in Itgax/eYFP/CD11c transgenic mice [44]. In this murine line, eYFP labels all CD11c+ cells, including Iba1+ cells, in the meninges and brain parenchyma [21], [44], [45]. In wholemount preparation of the dura mater, pia/arachnoid and choroid plexus, the entire population of eYFP/CD11c+ cells co-expressed Iba1 and MHCII [44]. Similar results were also obtained by immunophenotypical analyses on brain sections in CD11c diphtheria toxin receptor-GFP mice, raising the issue that fluorescent CD11c+ cells could represent a subclass of microglial cells rather than DCs [46]. This uncertainty does not apply to *thy1*GFP-M mice, in which no GFP+/Iba-1+ cells were identified, indicating that microglial cells do not express GFP in these mice.

Moreover, we found that, regardless of GFP expression, only a percentage of CD11c+ cells co-expressed MHCII (about 20% in the meninges and about 40% in the choroid plexus). It is possible that, as previously suggested [45], immunohistochemistry could be a less sensitive technique than genetic modification. On the other hand, gene insertion could be not entirely selective for a given cell population. As further confirmation of the identity of GFP+ cells as dendritic or monocyte-derived population of cells in *thy1*GFP-M mice, FACS analysis showed that these cell populations represent the highest percentage of GFP+ cells in whole blood.

### Conclusions

The present findings indicate that the *thy1*GFP-M mice provides a novel animal model for *in vivo* investigations of brain DCs. It has been recently stated that the recruitment of DCs into the brain by TPF microscopy remains to be explored [33]. The simultaneous visualization of fluorescently labeled neurons and DCs in *thy1*GFP-M mice could now allow this type of investigation as well as the study of other key aspects of neural-immune interactions.

## Supporting Information

Figure S1 **Comparison of fluorescence spectra of neuronal and non-neuronal cells in** ***thy1*** **GFP-M mice.** The plot shows the similarity of the fluorescence spectra acquired in neuronal cells (empty circle) and non-neuronal cells (full circle).
(TIF)

Figure S2 **Percentage of GFP positive cells in** ***thy1*** **GFP-M mouse blood cell populations.** Granulocytes (CD11b^+^ GR1^high^), monocytes (CD11b^+^ GR1^int^ F4/80^+^), dendritic cells (CD11b^+^ GR1^-^ F4/80^-^ CD11c^+^), B lymphocytes (B220^-^ CD3^-^), T cytotoxic cells (B220^-^ CD3^+^ CD8^+^ CD4^-^) and T helper (B220^-^ CD3^+^ CD8^-^ CD4^+^). Data are presented as means ± SD (n = 3).
(TIF)

Movie S1 **Motile non-neuronal GFP+ cell at pial level.** *In viv*o TPF microscopy movie of a non-neuronal GFP+ cell in the cortex of a *thy1*GFP-M mouse showing evident changes of shape over 55 minutes. On the top-right corner of the field, a faintly GFP+ moving cell is also visible. At minute 25 a fluorescent cell suddenly appears, suggesting a floating motion in the cerebrospinal fluid. Each frame is a maximum intensity projections of a set of optical sections acquired at 2 µm z-step.
(AVI)

Movie S2 **Non-neuronal GFP+ cell rolling inside a blood vessel.** *In vivo* TPF microscopy movie of a non-neuronal GFP+ cell (green) rolling inside a blood vessel (red). The vessel wall was visualized using the intravital dyeSR-101. Each frame is a maximum intensity projections of a set of optical sections acquired at 2 µm z-step.
(AVI)

Movie S3 **Translating non-neuronal GFP+ cell inside the brain parenchyma.** *In vivo* TPF microscopy movie of a non-neuronal GFP+ cell translating across the field. Each frame is a maximum intensity projections of a set of optical sections acquired at 2 µm z-step.
(AVI)

Movie S4 **Motile non-neuronal GFP+ cells observed through the thinned skull surgery.** *In vivo* TPF microscopy movie of non-neuronal GFP+ cells in a *thy1*GFP-M mouse in which the skull was thinned out (see section 2.2). GFP+ cells show the same motility observed in the open skull preparation, indicating that surgery did not affect the behavior of these cells. On the left, a GFP+ cell in a probing motion; in the center, a GFP+ cell in a scanning motion on the cortical surface; at minute 10, a GFP+ cell appears for one only frame and is probably floating in the cerebrospinal fluid. Each frame is a maximum intensity projections of a set of optical sections acquired at 2.5 µm z-step. Image depth: 73 µm from dura mater. Blood vasculature is visualized by iv injected tetramethyl rhodamine isothiocyanate-conjugated dextran.
(AVI)
