# Supplementary figures and images for: Two-Photon Microscopy Imaging of thy1GFP-M Transgenic Mice: A Novel Animal Model to Investigate Brain Dendritic Cell Subsets In Vivo
Source: PLoS One. 2013 Feb 7;8(2):e56144. doi: 10.1371/journal.pone.0056144 (PMC3567047; doi:10.1371/journal.pone.0056144)

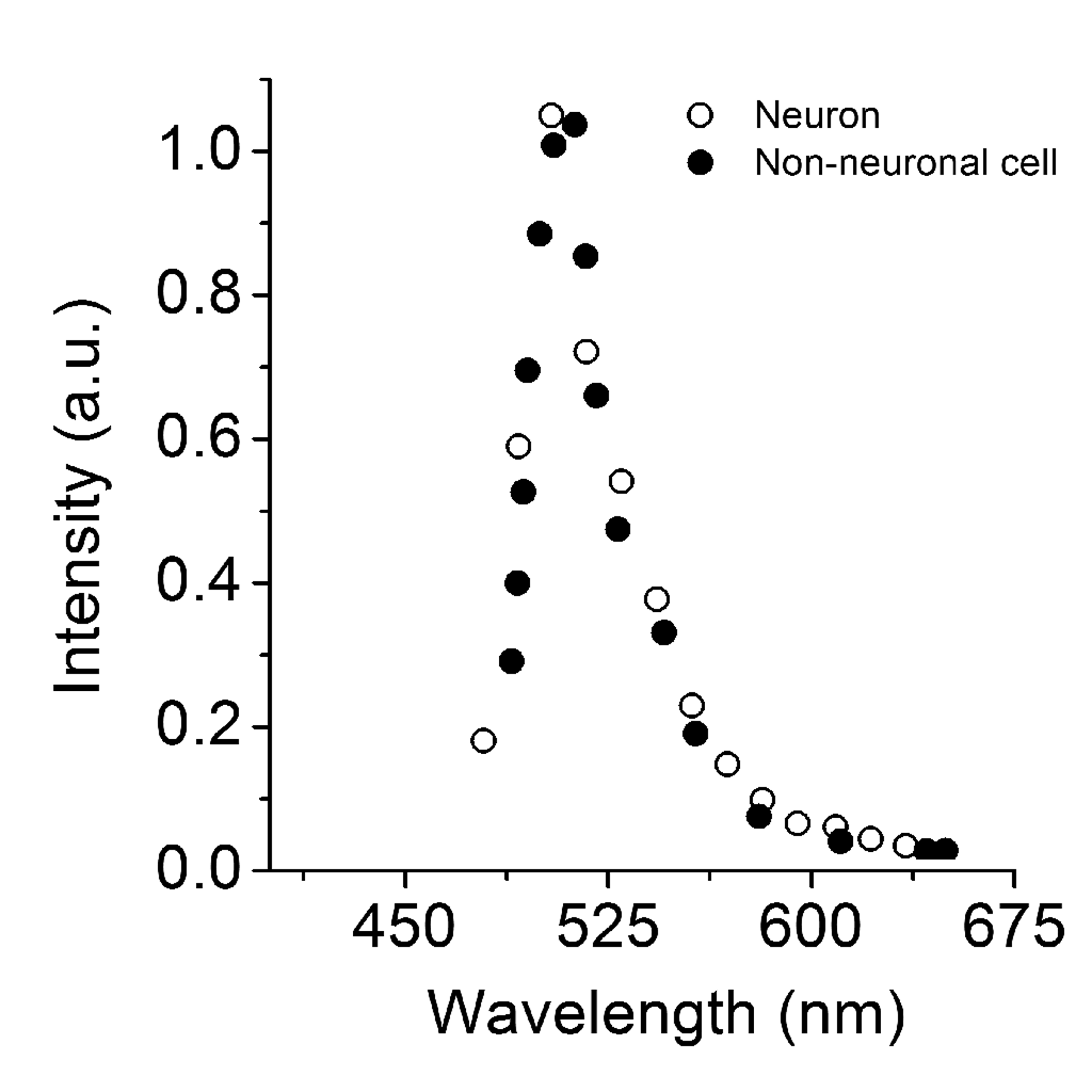

Supplement: Figure S1 — Comparison of fluorescence spectra of neuronal and non-neuronal cells in thy1 GFP-M mice. The plot shows the similarity of the fluorescence spectra acquired in neuronal cells (empty circle) and non-neuronal cells (full circle). (TIF) [file pone.0056144.s001.tif]

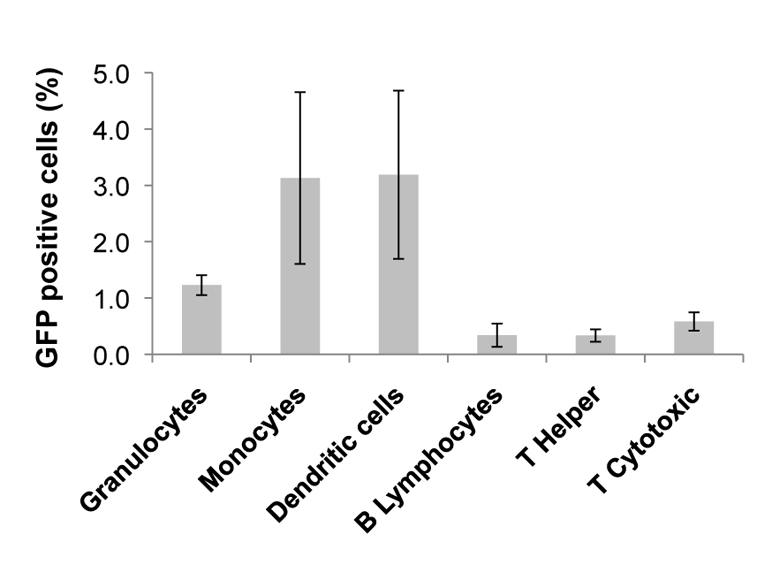

Supplement: Figure S2 — Percentage of GFP positive cells in thy1 GFP-M mouse blood cell populations. Granulocytes (CD11b+ GR1high), monocytes (CD11b+ GR1int F4/80+), dendritic cells (CD11b+ GR1- F4/80- CD11c+), B lymphocytes (B220- CD3-), T cytotoxic cells (B220- CD3+ CD8+ CD4-) and T helper (B220- CD3+ CD8- CD4+). Data are presented as means ± SD (n = 3). (TIF) [file pone.0056144.s002.tif]
